# A new family of aphids (Hemiptera, Sternorrhyncha) from the Lower Cretaceous of Baissa, Transbaikalia

**DOI:** 10.3897/zookeys.130.1444

**Published:** 2011-09-24

**Authors:** Agnieszka Homan, Piotr Wegierek

**Affiliations:** 1 Department of Zoology, University of Silesia, Bankowa 9, 40-007 Katowice, Poland

**Keywords:** Hemiptera, aphids, fossil insects, Lower Cretaceous, Baissa, new family, new genus, new species

## Abstract

The family Rasnitsynaphididae **fam. n.** has a unique combination of characters: 9-segmented antennae; rhinaria arranged in many transverse rows, surrounding the antennal segments; segment IX narrower than other segments of flagellum, always without rhinaria; cubitus branches separated; ovipositor present; siphuncular pores absent. The new family comprises the genus *Rasnitsynaphis* **gen. n.** with three species, *Rasnitsynaphis ennearticulata* **sp. n.**, *Rasnitsynaphis coniuncta* **sp. n.**, and *Rasnitsynaphis quadrata* **sp. n.,** all from the Lower Cretaceous of Transbaikalia.

## Introduction

Baissa is one of the most important Lower Cretaceous insect fossil sites. The outcrop is located in the Asian part of Russia, in western Transbaikalia, on the left bank of the Vitim River. The exact age of lacustrine sediments of the Zaza Formation at Baissa is disputable, usually estimated as Early Cretaceous (Neocomian–Aptian), however most paleoentomologists date them as Valanginian–Hauterivian (Zherikhin et al. 1999).

More than 20 000 fossil insects (including above 2 500 aphid specimens), often of excellent preservation state, have been collected there. Nearly all aphids from Baissa belong to three families: Ellinaphididae Kania & Wegierek, 2008, Szelegiewicziidae Wegierek, 1989 (both in Palaeoaphidoidea), and Oviparosiphidae Shaposhnikov, 1979 (Aphidoidea). The new taxa described below show an even higher morphological diversity of aphids in the Lower Cretaceous.

## Material and methods

The material comes from the collection of the Laboratory of Arthropods, Paleontological Institute (PIN), Russian Academy of Sciences, Moscow, where it is currently stored. The research methods did not differ substantially from those widely used in paleoentomological research (Rasnitsyn 2002). The imprints were photographed under the stereoscopic and the light microscope. The graphics tablet was applied to make the drawings on the photographic layer in Adobe Photoshop. In case when the specimen was represented by two imprints, the drawings are based on both reverse and obverse, while the photograph presents only one imprint. The number of rhinaria given in the descriptions always relates only to the one side of the antenna. It results from the lack or the weak preservation of one of the imprints. All measurements are given in mm.

## Taxonomy

### Hemiptera Linnaeus, 1758

**Sternorrhyncha Amyot & Serville, 1843**

**Aphidomorpha Becker-Migdisova & Aizenberg, 1962**

#### Rasnitsynaphididae fam. n.

urn:lsid:zoobank.org:act:94C0708C-7F79-426A-B9FB-1DD3D53BAF67

http://species-id.net/wiki/Rasnitsynaphididae

##### Type genus.

*Rasnitsynaphis* gen. n.

##### Diagnosis.

Antennae 9-segmented, shorter than hind tibia. Rhinaria arranged in many transverse rows, surrounding antennal segments. Segment IX narrower than other segments of flagellum, blunt at apex, always without rhinaria. Cubitus branches separated. Vein Rs separates from pterostigma in one third of its length. Media originates below the base of pterostigma. Ovipositor present. Siphuncular pores absent.

#### Rasnitsynaphis gen. n.

urn:lsid:zoobank.org:act:2466FD65-63B1-4F19-9BC1-576C84461B21

http://species-id.net/wiki/Rasnitsynaphis

##### Type species.

*Rasnitsynaphis ennearticulata* sp. n.

##### Etymology.

Named to honour Alexandr P. Rasnitsyn, who is not only eminent researcher, but also a teacher of many paleoentomologists. P.W. is honoured to be one of his students.

##### Diagnosis.

As for family.

##### Description.

Body massive. Front edge of head convex. Head with epicranial suture. Rostrum shorter than body, reaching to the middle of abdomen. Antennae longer than one third of the body length but shorter than its half. Antennal segment III 2–4 times as long as wide; segments IV-VIII of the same length or nearly so. Surface of all segments of flagellum sculptured in form of transverse ribs. Rhinaria ellipsoidal, arranged in more or less dense rows which are sometimes joined. Cubital vein CuA1 2.5 times longer than CuA2. Pterostigma big, spindle-shaped, pointed, 3–3.5 times as long as wide. Vein Rs slightly bent at base. Media with two or three branches. Bifurcation of vein M into M1 and M2 or into M1+2 and M3 just beyond the base of vein Rs.

##### Key to species of *Rasnitsynaphis*

**Table:** 

1	Antennae short, about 1/2 of thorax height, segment III two times as long as wide, with ca. 9 rows of rhinaria; segments IV–VIII as long as wide; M with 2 branches	*Rasnitsynaphis quadrata* sp. n.
–	Antennae longer, segment III three to four times as long as wide; segments IV–VIII longer than wide	2
2	Segment III four times as long as wide, with ca. 11 rows of rhinaria	*Rasnitsynaphis coniuncta* sp. n.
–	Segment III three times as long as wide, with ca. 17 rows of rhinaria; M with 3 branches	*Rasnitsynaphis ennearticulata* sp. n.

#### Rasnitsynaphis ennearticulata sp. n.

urn:lsid:zoobank.org:act:9040D3E2-3EFE-474D-889A-2408FD52A5BC

http://species-id.net/wiki/Rasnitsynaphis_ennearticulata

Fig. 1

##### Material.

Holotype: PIN 3064/2109(2211); Baissa, Transbaikalia; Zaza Formation, bed 31.

##### Etymology.

From the Greek term *ennea* for “nine” and from the Latin term *articulum* for “segment” in reference to the 9-segmented antennae.

##### Diagnosis.

Antennae rather long; segment III three times as long as wide;segments IV–VIII of about the same length, rectangular, longer than wide. Media with three branches.

##### Description.

**Figure 1.**
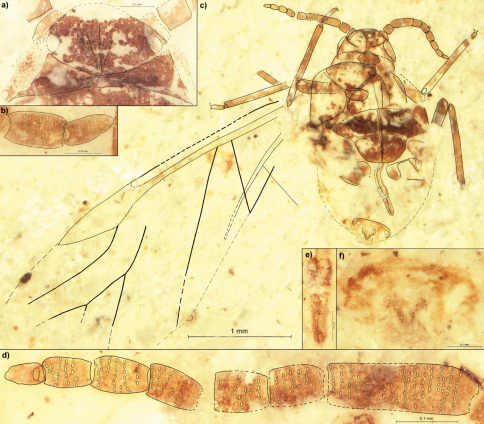
*Rasnitsynaphis ennearticulata* sp. n., PIN 3064/2109(2211) (holotype): **a** head, dorsal view **b** VIII and IX segment of right antenna, ventral view **c** body, ventral view **d** flagellum of left antenna, ventral view **e** apical segment of rostrum, ventral view **f** apical part of abdomen with ovipositor, ventral view.

Length of the body 2.1 (Fig. 1c). Width of head 0.43 (Fig. 1a). Lateral sutures join in the middle of epicranium in the four fifth of the head length. On the dorsal side of head capsule there are three diagonal strips, running from the middle part of epicranium to the frontolateral edge of head. The distance between ocelli (situated on the back of head) 0.27. Length of the apical segment of rostrum 0.18 (Fig 1e). Antennae 0.93 long, ca. 0.8 of thorax width (Figs 1b, d). Length of antennal segments: I 0.06; II 0.08; III 0.23; each of IV–VII 0.09; VIII 0.08; IX 0.07. Antennal segment III with about 17 rows of rhinaria, with at most 8 rhinaria arranged in one row. Segments IV–VIII at most with 7 rows of rhinaria. Tibia of fore legs 0.75 long, middle tibiae 0.83 to 0.86. Mesothoracic sternite 0.93 wide, 0.53 long. Fore wings about 3.8 long. The distance from the base of the wing to the end of pterostigma 2.8. Distance between bases of cubital veins CuA1 & CuA2 0.14. The length of M1+2 more or less equals the length of the common stem of M. The posterior part of abdomen strongly sclerotized (Fig. 1f).

#### Rasnitsynaphis coniuncta sp. n.

urn:lsid:zoobank.org:act:197A9263-DFAA-4A61-9774-822FE7D3585D

http://species-id.net/wiki/Rasnitsynaphis_coniuncta

Fig. 2

##### Material.

Holotype: PIN 3064/2209; Baissa, Transbaikalia; Zaza Formation, bed 31.

##### Etymology.

From the Latin term *coniunctus* for “joined” in reference to the joined rows of rhinaria.

##### Diagnosis.

Antenna rather long; segment III four times as long as wide; segments IV–VIII of about the same length, rectangular, longer than wide.

##### Description.

**Figure 2.**
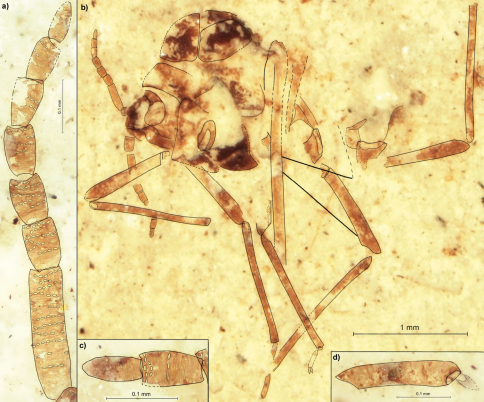
*Rasnitsynaphis coniuncta* sp. n.*,* PIN 3064/2209 (holotype): **a** flagellum of right antenna **b** body, lateral view **c** VIII and IX segment of left antenna **d** second segment of hind tarsus.

Length of the body about 2.4 (Fig. 2b). Head with epicranial suture. Antennae 0.88 long, about 2/3 of thorax height (Figs 2a, c). Length of antennal segments: II 0.12; III 0.24; IV 0.09; V 0.08; each of VI–VII 0.09; VIII 0.08; IX 0.07 to 0.08. Antennal segment III with 11 rows of rhinaria, with at most 7 rhinaria arranged in one row. Segments IV–VIII at most with 6 rows of rhinaria. Femur of fore legs 0.74 long, tibia 1.06. Middle tibia 1.14 long. Hind femur 0.87 long, tibia 1.34. The second segment of hind leg tarsus 0.24 (Fig. 2d). The distance between bases of cubital veins CuA1 & CuA2 0.13.

#### Rasnitsynaphis quadrata sp. n.

urn:lsid:zoobank.org:act:7434FB55-DE6B-4C54-9086-F2E62111405D

http://species-id.net/wiki/Rasnitsynaphis_quadrata

Fig. 3

##### Material.

Holotype: 3064/2279; Baissa, Transbaikalia; Zaza Formation, bed 31.

##### Etymology.

From the Latin term *quadratus* for “square” in reference to the square shape of antennal segments IV–VII.

##### Diagnosis.

Antennae quite short; segment III two times as long as wide; segments IV–VII of the same length, square, as long as wide. Media with two branches.

##### Description.

**Figure 3.**
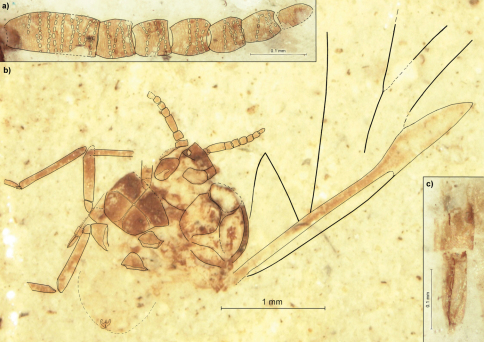
*Rasnitsynaphis quadrata* sp. n., PIN 3064/2279 (holotype): **a** flagellum of right antenna, dorsal view **b** body, ventral view **c** apical segment of rostrum.

Length of the body 2.0 (Fig. 3b). Length of head 0.28. Head with lateral sutures. Length of the apical segment of rostrum 0.14 (Fig. 3c). Antennae 0.71 long, about 1/2 of thorax height (Fig. 3a, b). Length of antennal segments: I 0.06; II 0.07; III 0.16; each of IV–IX 0.06. Antennal segment III with about 9 rows of rhinaria, with at most 8 rhinaria arranged in one row. Segments IV–VIII at most with 4 rows of rhinaria. Femur of middle legs 0.54 long, tibia 0.73. Hind femur 0.57 long. Mesothoracic sternite 0.49 long. The distance from the base of the wing to the end of pterostigma 2.7. The distance between bases of cubital veins CuA1 & CuA2 0.11. The posterior part of abdomen weakly sclerotized.

## Discussion

The family Rasnitsynaphididae fam. n. on the one hand possesses primitive features of Jurassic Sinojuraphididae Huang & Nel, 2008 and Genaphididae Handlirsch, 1907, but on the other hand the features typical for Early Cretaceous Oviparosiphidae and Ellinaphididae, and also certain features of recent Aphididae and Drepanosiphidae (Heie and Wegierek 2009).

The body morphology and wing venation of the new family are typical for Lower Cretaceous aphids. The 9-segmented antennae recorded in Rasnitsynaphididae is the plesiomorphic feature and proves the presence of forms intermediate between Middle Jurassic Sinojuraphididae with 12-segmented antennae (Huang and Nel 2008) and Mesozoic and modern aphids with 7-, 6- or 5-segmented antennae. In the general structure of antenna, which is relatively short and massive, it resembles many representatives of Oviparosiphidae. Due to the arrangement of numerous small, ellipsoidal rhinaria in transverse rows, Rasnitsynaphididae is similar to Ellinaphididae and *Jurocallis* Shaposhnikov, 1979, however the length ratio of flagellum segments is different.

In the shape of pterostigma and place of Rs separation *Rasnitsynaphis* is similar to the species of *Oviparosiphum* Shaposhnikov & Wegierek, 1989. In the structure of CuA and M veins *Rasnitsynaphis* resembles *Bajsaphis* Shaposhnikov, 1985, *Acanthotrichaphis* Shaposhnikov & Wegierek, 1989, *Vitimaphis* Shaposhnikov & Wegierek, 1989, and other genera of the family Oviparosiphidae (Zhang et al. 1989). In the course of the M vein *Rasnitsynaphis ennearticulata* sp. n. is similar also to *Penaphis* Lin, 1980 (Jarzembowski 1989, Lin 1995) and some species of the families Aphididae and Drepanosiphidae (Shaposhnikov 1980).

Having so many features in common with the family Oviparosiphidae, the new family differs from it by the 9-segmented antennae and lack of siphunculi.

## Supplementary Material

XML Treatment for Rasnitsynaphididae

XML Treatment for Rasnitsynaphis

XML Treatment for Rasnitsynaphis ennearticulata

XML Treatment for Rasnitsynaphis coniuncta

XML Treatment for Rasnitsynaphis quadrata
